# ATP-Binding Cassette Transporters in the Clinical Implementation of Pharmacogenetics

**DOI:** 10.3390/jpm8040040

**Published:** 2018-12-05

**Authors:** Luis A. López-Fernández

**Affiliations:** 1 Pharmacy Department, Instituto de Investigación Sanitaria Gregorio Marañón, Hospital General Universitario Gregorio, 28007 Madrid, Spain; luis.lopez@iisgm.com; 2 Spanish Clinical Research Network (SCReN), 28040 Madrid, Spain

**Keywords:** pharmacogenomics, ATP-binding cassette, adverse drug reactions, drug efficacy

## Abstract

ATP-binding cassette (ABC) transporters are involved in a large number of processes and contribute to various human genetic diseases. Among other functions, ABC proteins are involved in the transport of multiple drugs through cells. Most of the genes coding for these transporters are highly polymorphic and DNA variants in these genes can affect the normal functioning of these proteins, affecting the way drugs are transported, increasing or decreasing drug levels. These changes in the intracellular and extracellular drug levels may be associated with altered drug effectiveness or severe drug-induced adverse events. This review presents a state-of-art of the most pharmacogenetics clinically relevant ABC transporters closed to the clinical implementation.

Although many authors identify DNA variants that affect the way an individual patient can respond to a drug or suffer severe adverse reactions, few of these variants have shown a high level of evidence and fewer still have sufficient evidence to be implemented in clinical practice. This statement for pharmacogenetics in general is also valid for ATP-binding cassette (ABC) transporters in particular. After a brief summary of ABC transporters and an introduction to pharmacogenetics, this review will delve into the variants of ABC transporters with a currently high or moderate level of evidence to be used in clinical practice. Since the level of evidence is sometimes a fuzzy line, this paper has considered the classification of clinical annotations in ABC transporters variants on the most reputable non-profit organization in pharmacogenetics, PharmGKB, and the guidelines of the Clinical Pharmacogenomics Implementation Consortium (CPIC). The most important genes containing these variants and the works on which this classification is based are presented. Finally, some ways to improve the clinical implementation of ABC-transporter pharmacogenetics are suggested.

## 1. ABC Transporters

ATP-binding cassette (ABC) transporters are a family of ATP-dependent proteins involved in a large number of processes that contribute to various human diseases, such as cardiovascular diseases, ulcerative colitis, or Alzheimer [1,2,3,4]. They also transport endogenous and exogenous molecules and regulate cell integrity, metabolism and homeostasis. They have a major role in the transport of a large number of drugs used in many diseases and are involved in their efficacy and toxicity. For this reason, changes in the level of expression and functionality of these transporters influence the efficacy and safety of the drugs transported. The study of ABC transporters in drug development is also critical [5]. Among ABC transporters, multidrug resistance proteins are known to contribute significantly to multidrug-resistant cancer [6], but many other members have also demonstrated their relevance in interindividual variability response to drugs in many other diseases [7,8].

There are 48 known human ABC transporters, which are classified into 6 subfamilies (ABCA, ABCB, ABCC, ABCD, ABCE/ABCF and ABCG). ABC transporters have four domains: two nucleotide-binding domains (NBDs) and two transmembrane domains (TMDs). The NBDs bind and hydrolyze ATP, while the TMDs recognize and transport substrates [9]. The ABCA family contains some of the largest transporters that have been linked primarily to lipid trafficking [10]. The ABCB family contains four full and seven half transporters, some of them located in the blood-brain barrier, liver or mitochondria. They are involved in the transport of bile and peptides [11]. ABCC transporters contain 13 full molecules, including the cystic fibrosis transmembrane conductance regulator (CFTR) protein, also known as ABCC7, which causes cystic fibrosis; cell-surface receptors, such as the sulfonylurea receptors (ABCC8, ABCC9) and the multidrug resistance proteins (MRPs) [12]. ABCC proteins are mostly involved in the transport of endo- and xenobiotics. The ABCD family contains only four members and all of them are used in peroxisomes [13]. ABCE and ABCF proteins are often considered as a single family and are not transporters. They maintain the ATP-domain but lack the transmembrane domain and are involved in the expression and regulation of protein synthesis [9]. Finally, the ABCG family consist of six half-transporters involved in the transport of lipids, drug substrates, bile, cholesterol, and other steroids [14]. However, not all of these ABC transporter genes have shown to be relevant in the way patients respond to drugs.

## 2. Pharmacogenetics and Clinical Evidence: What about ABC Transporter Variants

Pharmacogenetics is defined by the food and drug administration (FDA) and European European Medicines Agency (EMA) as the study of variations in DNA sequence as related to drug response while pharmacogenomics includes pharmacogenetics as well as study the variations of RNA characteristics as related to drug response, (http://www.ema.europa.eu/docs/en_GB/document_library/Scientific_guideline/2009/09/WC500002880.pdf). These disciplines do not include others such as proteomics and metabolomics, but they do include processes such as drug absorption and disposition, drug effects, drug efficacy and adverse effects of drugs. There are many well-stablished relations between DNA variants in genes involved in pharmacodynamics and pharmacokinetics of drugs. The international transporter consortium has recently published a review of the influence of transporter polymorphisms on drug availability and response [15]. Although this paper reviews a large number of associations between polymorphisms in ABC transporters and drug response with a high statistically significant value, the clinical utility of these variants is in doubt.

In PharmGKB, the level of evidence of the clinical annotation of a DNA variant with the toxicity or efficacy of a drug is classified from 1 to 4. Level 1 corresponds to high evidence, level 2 to moderate evidence, level 3 to low evidence and level 4 to preliminary evidence. Categories 1 and 2 are also subdivided into levels, A and B, the former being more significant than the latter. Thus, level 1A is assigned to annotations for a variant-drug combination in a guideline or implemented at a major health system. Level 1B includes annotations with high evidence, but the association variant-drug must be replicated in more cohorts with significant *p*-values and with a strong effect size. Level 2 is also divided into A and B. Both have a moderate evidence, but in the case of 2A it corresponds to “very important pharmacogenes” as defined by PharmGKB [16]. Regarding ABC-transporter genes, 14 of them with clinical annotations related to drug response or adverse events have been included in PharmGKB: *CFTR*, *ABCA1, ABCB5, ABCB1, ABCC4, ABCC5, ABCC1, ABCC1, ABCC2, ABCC3, ABCC6, ABCC10, ABCG2* and *ABCG1*. DNA variants in *CFTR* and ivacaftor treatment are the only ABC transporters associations to reach level 1A or 1B and currently implemented in clinical pharmacogenetics [17]. In the Table 1, a summary of associations from a level 1A to 2B is introduced.

Furthermore, The Clinical Pharmacogenetics Implementation Consortium (CPIC), the Royal Dutch Association for the Advancement of Pharmacy (DPWG), the Canadian Pharmacogenomics Network for Drug Safety (CPNDS) and other societies have made a great effort in this regard and have promoted the publication of dozens of guidelines. However, although many ABC transporters variants have been studied, no specific clinical recommendations are currently given, except for CFTR [18,19].

## 3. ABC Transporter Genes with High and Moderate Evidence of Clinical Implementation

Only 4 ABC transporters genes, as shown in Table S1, have level 1 or 2 evidence of clinical annotations. A detailed description of the works on which this classification is based is presented below.

### 3.1. CFTR

CTFR functions as an ion channel and controls ion and water secretion and absorption in epithelial tissues, but it does not function as a transporter. CFTR is the only ligand-gated channel that consumes its ligand (ATP) during the gating cycle [59]. Malfunction of this channel causes cystic fibrosis, a disorder that affects the production of sweat, digestive fluids and mucus causing difficulty breathing, and coughing up mucus as a result of lung infections [60]. Ivacaftor drug label indicates its use in patients diagnosed with cystic fibrosis with at least one copy of any of a group of *CFTR* genetic variants. The variants required for genotyping prior to prescribing ivacaftor change according to regulatory agencies. For instance, the FDA includes 33 DNA variants, while the EMA includes only nine of them (see Table 2). All these DNA variants produce mutant CFTR forms with mild defects in CFTR processing or channel conductance [20]. This was first observed for rs11971167, which produces a change from an Asp to an Asn at position 1270 of CFTR. This change is responsible for a 1.9-fold increase in chloride transport after ivacaftor treatment compared to baseline (no ivacaftor-treatment).

### 3.2. ABCB1

MDR1 or ABCB1 is an efflux transporter involved in the transport of multiple drugs and many processes. There is an important interindividual variability that can be explained mainly by genetic variants [61]. Three polymorphisms have been extensively studied, two synonymous SNPs, 1236C > T, (rs1128503) in exon 12 and 3435C > T (rs1045642) in exon 26; and one nonsynonymous SNP, 2677G > T/A (rs2032582 in exon 21). These three polymorphisms are in linkage disequilibrium and define, among others, the haplotype ABCB1*2. This haplotype is related to an increased activity in MDR1 [62]. Nevertheless, conflicting data of these polymorphisms make it difficult to apply them in clinical practice [63,64,65].

Variants in *ABCB1* have been related to hundreds of drugs. However, only a few of them reach a level 2 of evidence in PharmGKB. The variants rs1128503 (1236C > T), rs2032582 (2677G > A/T) and rs1045642 (3435C > T) in *ABCB1* have been associated with efficacy to simvastatin in men [21,22]. Since the three most studied SNPs in *ABCB1* are in linkage disequilibrium, many authors have attempted to assign pharmacogenetic associations to the haplotypes defined by them. In this sense, two haplotypes are the most relevant: *1 with the genotype CGC and *2 with the genotype TTT. For instance, Becker and col. found that men **simvastatin** users with the 1236/2677/3434 TTT and CGT haplotypes had larger reductions in total cholesterol and low-density lipoprotein cholesterol compared to the wild-type CGC haplotype [7]. Severe adverse reactions induced by simvastatin are also associated with *ABCB1* polymorphisms. Thus, individuals carrying TTT haplotype developed less myalgia during simvastatin treatment than non-carriers [21]. However, a more recent study shows that these *ABCB1* variants have no effect on simvastatin pharmacokinetics in Korean men [66]. This discrepancy may be responsible for not reaching a level 1 of evidence.

These variants in *ABCB1* have also been associated with response or toxicity to other drugs. Thus, the allele T for rs1045642 is associated with **methotrexate**-induced toxicity with a level 2A. Therefore, the TT and CT genotypes for the rs1045642 and rs1128503 variants in *ABCB1* were associated with severe neutropenia in children with acute lymphoblastic leukemia [23]. These authors also showed that the polymorphism rs717620 *ABCC2*, another ABC efflux transporter, was associated with low methotrexate levels. However, the evidence for variant rs1045642 and methotrexate-induced toxicity is greater because other associations were also found between the T allele and hepatic toxicity [67], anemia and thrombocytopenia [68]. Most of these works did not genotyped the polymorphisms rs1128503 and rs2032582, which are in linkage disequilibrium with rs1045642. For this reason, these other two *ABCB1* polymorphisms may also be associated with methotrexate-induced toxicity. However, further studies are needed to clarify this issue. 

The variant rs1045642 in *ABCB1* is also associated with **nevirapine**-induced toxicity. Patients with the TT genotype for this variant may have a decreased, but not absent, risk for nevirapine-induced hepatotoxicity compared to patients with the CC genotype [24,25,26]. The *ABCB1* *2/*2 diplotype was associated with a decrease in the clearance of **atazanavir** when taken alone or co-administrated with ritonavir, as well as of ritonavir taken alone compared to the *1/*1 or *2/*1 diplotypes [27,28].

Two variants of *ABCB1* associated with toxicity and/or efficacy for **ondansetron** have been found with a level of evidence 2A. Variant rs1045642 is associated with likelihood of postoperative nausea and vomiting in patients with acute leukemia when treated with the antiemetic ondansetron [29,30]. Thus, patients with AA genotype suffer less nausea and vomiting than patients with CT or CC.

Variant rs1045642 has also been associated with the metabolism of **digoxin** with a level 2A. Patients carrying the allele T had higher serum concentrations, higher bioavailability, and less renal clearance of digoxin compared to those patients carrying the C allele [31,32,33]. 

Drugs that inhibit or induce *ABCB1* expression, as well as DNA variants, can alter the efflux of blood-brain barrier and affect the efficacy of many drugs, such as **opioids** [69]. The rs1045642 is related to the dose and efficacy of **fentanyl, methadone, morphine, opioids, oxycodone or tramadol** with an 2B evidence level. Patients with the AA genotype may experience improved opioid efficacy and may require a lower dose compared to patients with GG genotype and possible AG. However, the results are contradictory. Thus, some authors found a relationship between dose requirements for any of these drugs and pain [34,35,36,37,38], but many others did not [39,40,41,42,43,44]. Other authors even found an opposite relationship, showing how patients with genotype AA require a higher maintenance dose of methadone than patients with genotypes GG or AG [70].

Finally, another association between *ABCB1* variants and drug response with an evidence level 2B refers to **sunitinib**. Patients with renal cell carcinoma and the haplotype *ABCB1* *2/*2 may show a decreased response to sunitinib compared to other patients. Decreased overall survival and progression-free survival were showed along with a reduced risk of neutropenia [71,72].

### 3.3. ABCC4 and ABCG2

ABCC4, also known as MRP4, transports many xenobiotics and is expressed in organs and cells relevant for drug delivery, such as liver, kidneys and blood cells. The structural pattern of this transporter and the effect of some polymorphisms on it has recently been reported [73]. Similarly, some variants have shown to differentially modulate the transport of methylated arsenic metabolites and physiological organic anions [74]. In terms of major clinical associations of *ABCC4*, the TT genotype for rs1751034 SNP in *ABCC4* has been associated with increased **tenofovir** renal clearance and a lower intracellular tenofovir diphosphate in HIV patients [45,46]. The lack of further research and the low but significant *p*-value (0.4–0.5) may be responsible for having only a level 2B of evidence.

ABCG2, also known as BCRP, MXR or MCF-7, is an ABC half transporter involved in the transport or substances such as chemotherapeutics, antivirals, antibiotics, and flavonoids [75]. Several *ABCG2* DNA variants, such as Phe489Leu (c.1465 T> C) and Tyr469Ala have been shown to reduce its protein expression by affecting the effect of drugs that interacts with ABCG2 [76]. However, another variant, rs2231142 (Gln141Glu), is the most relevant for the pharmacogenetics of *ABCG2*. The T allele of this SNP is associated with a decrease in **allopurinol** response in patients with gout. People with GG genotype may have a better response when treated with allopurinol and may require a lower dose compared to patients with the GT or TT genotypes [47,48].

Unlike patients taking allopurinol, those taking **rosuvastatin** and carrying the T allele for rs2231142 SNP in *ABCG2* may have a higher plasma concentration of the drug and a greater response determined by a higher reduction in low-density lipoprotein cholesterol in hypercholesterolemia and myocardial infarction [50,51,52,53,54,55,56,57,58]. These associations have a level of evidence 2B in PharmGKB.

## 4. Ways to Improve Clinical Implementation of ABC Transporters Pharmacogenetics

Current knowledge of the pharmacogenetics of ABC transporters can be improved in three different ways: first, by studying more known variants; second, by using genome-wide techniques that can also discover new variants; and third, by increasing the evidences already stablished.

As for the first point, searching for potentially relevant DNA variants in ABC transporter genes in the Exome Aggregation Consortium (ExAC) browser (http://exac.broadinstitute.org/) may give us a view of possible polymorphisms to study in future work [77]. Supplemental Table S1 presents a search for variants in ExAC with a minor allele frequency higher than 1% in a general population of 60,706 humans and located in coding or regulatory regions (5′UTR, 3′UTR or splice regions) in ABC transporter genes with clinical annotations in PharmGKB. Among these variants, we find the most studied SNPs in pharmacogenetics of ABC transporters. However, not all of these variants have been included in previous studies, which opens the door to their selection and analysis as good candidates in the future.

The second way is the use of next generation sequencing and genome-wide association studies. This type of analysis is also contributing and will contribute to increasing the number of known pharmacogenetic variants in ABC transporters [78].

Probably, the quickest option to improve the clinical implementation of ABC-transporter pharmacogenetics is to increase studies in variants with level of evidence 1B, 2A and 2B. The lack of strong evidence of association of genetic variants with drug response has been one of the main reasons why clinical implementation of pharmacogenetics has experienced many difficulties [79]. Even, when solid evidence is found, its clinical usefulness is often questioned.

All these ways together are the best options to generate guidelines to help for the clinical implementation of ABC transporters pharmacogenetics.

## 5. Conclusions and Perspectives

Obviously, there are many more studies relating DNA variants in ABC-transporter genes associated with drug response or toxicity than those shown in this review. However, these associations do not have a high evidence level because most of the time results are contradictory. There are findings showing the association of a variant with a specific drug response or induced-toxicity, and others with the same credibility that show the lack of association, or worse, that show the same association but with the opposite allele. For instance, the T alleles in *ABCB1* variants rs1045642, rs1128503 and rs2032582 SNPs have been associated with both a lower and a higher risk of severe irinotecan-induced toxicity [80,81,82]. Examples like this are very common in pharmacogenetics in general, and in ABC-transporters in particular.

Nevertheless, the successful example of *CFTR* variants and ivacaftor treatment, and the promising biomarkers such as those rated as level 2A and B by PharmGKB, allow us to be optimistic in the future. We are required to rise knowledge of these 2A–2B biomarkers to advance clinical implementation. In addition, the expected amount of information that come with massive sequencing programs under development in several countries could boost pharmacogenetic knowledge of ABC transporters. Finally, future meta-analysis and clinical trials could help further develop the implementation of ABC-transporters pharmacogenetics.

## Figures and Tables

**Table 1:** ATP-binding cassette (ABC) transporter variants with a level of evidence from 1A to 2B.

Level	Variant	Gene	Drug	Effect	PMID
1A	rs11971167	*CFTR*	Ivacaftor	Efficacy	23891399 [20]
2A	rs2032582	*ABCB1*	Simvastatin	Efficacy	16321621 [21], 19891551 [22]
2A	rs1045642	*ABCB1*	Methotrexate	Toxicity	25007187 [23]
2A	rs1045642	*ABCB1*	Nevirapine	Toxicity	20017669 [24], 16912956 [25], 16912957 [26]
2A	ABCB1*1, ABCB1*2	*ABCB1*	Atazanavir	Toxicity/PK	19710077 [27] 22394315 [28]
2A	rs2032582 rs1045642	*ABCB1*	Ondansetron	Efficacy	20707787 [29] 25012726 [30]
2A	rs1045642	*ABCB1*	Digoxin	Others	18334914 [31], 12189368 [32], 10716719 [33]
2B	rs1045642	*ABCB1*	Fentanyl, methadone, morphine, opioids, oxycodone, tramadol	Dosage/efficacy	[34,35,36,37,38,39,40,41,42,43,44]
2B	ABCB1*1, ABCB1*2	*ABCB1*	Sunitinib	Efficacy	
2B	rs1751034	*ABCC4*	Tenofovir	Pk	17597712 [45], 18398970 [46]
2B	rs2231142	*ABCG2*	Allopurinol	Dosage/efficacy	25676789 [47], 26810134 [48], 29341237 [49]
2B	rs2231142	*ABCG2*	Rosuvastatin	Efficacy	20130569 [50], 20679960 [51], 16784736 [52], 19474787 [53], 23930675 [54], 23930675 [54], 23876492 [55], 25630984 [56], 20207952 [57], 28322941 [58]

**Table 2:** *CFTR* DNA variants included in the drug label by different drug regulatory agencies.

Amino-Acid Change	SNP ID	FDA	EMA	HCSC
Gly56Lys	rs397508256	✓		
Pro67Leu	rs368505753	✓		
Arg74Trp	rs115545701	✓		
Asp110Glu	rs397508537	✓		
Asp110His	rs113993958	✓		
Arg117Cys	rs77834169	✓		
Arg117His	rs78655421	✓		
Gly178Arg	rs80282562	✓	✓	✓
Glu193Lys	rs397508759	✓		
Leu206Trp	rs121908752	✓		
Arg347His	rs77932196	✓		
Arg352Gln	rs121908753	✓		
Ala455Glu	rs74551128	✓		
Ser549Asn	rs121908755	✓	✓	✓
Ser549Arg	rs121908757, rs121909005	✓	✓	✓
Gly551Asp	rs75527207	✓	✓	✓
Gly551Ser	rs121909013	✓	✓	✓
Asp579Gly	rs397508288	✓		
Ser945Leu	rs397508442	✓		
Gly970Arg	rs397508453			✓
Ser977Phe	rs141033578	✓		
Phe1052Val	rs150212784	✓		
Lys1060Thr	rs397508513	✓		
Ala1067Thr	rs121909020	✓		
Gly1069Arg	rs200321110	✓		
Arg1070Gln	rs78769542	✓		
Arg1070Trp	rs202179988	✓		
Phe1074Leu	rs186045772	✓		
Asp1152His	rs75541969	✓		
Gly1244Glu	rs267606723	✓	✓	✓
Ser1251Asn	rs74503330	✓	✓	✓
Ser1255Pro	rs121909041	✓	✓	✓
Asp1270Asn	rs11971167	✓		
Gly1349Asp	rs193922525	✓	✓	✓

SNP: single nucleotide polymorphisms; EMA: European Medicines Agency; FDA: Food and drug administration; HCSC: Health Canada (Santé Canada).

## Supplementary Materials

The following are available online at http://www.mdpi.com/2075-4426/8/4/40/s1, Table S1: Main variants in the ABC transporter genes with the greatest potential interest in pharmacogenetics.
